# Comparison of Lung-Homing Receptor Expression and Activation Profiles on NK Cell and T Cell Subsets in COVID-19 and Influenza

**DOI:** 10.3389/fimmu.2022.834862

**Published:** 2022-03-16

**Authors:** Demi Brownlie, Inga Rødahl, Renata Varnaite, Hilmir Asgeirsson, Hedvig Glans, Sara Falck-Jones, Sindhu Vangeti, Marcus Buggert, Hans-Gustaf Ljunggren, Jakob Michaëlsson, Sara Gredmark-Russ, Anna Smed-Sörensen, Nicole Marquardt

**Affiliations:** ^1^Center for Infectious Medicine, Department of Medicine Huddinge, Karolinska Institutet, Stockholm, Sweden; ^2^Department of Infectious Diseases, Karolinska University Hospital, Stockholm, Sweden; ^3^Division of Infectious Diseases, Department of Medicine Huddinge, Karolinska Institutet, Stockholm, Sweden; ^4^Department of Medicine Solna, Karolinska Institutet, Stockholm, Sweden; ^5^Division of Immunology and Allergy, Department of Medicine Solna, Karolinska Institutet, Karolinska University Hospital, Stockholm, Sweden

**Keywords:** NK cells, T cells, chemokine receptors, lung-homing, SARS-CoV-2, COVID-19, influenza

## Abstract

Respiratory viral infections with SARS-CoV-2 and influenza viruses commonly induce a strong infiltration of immune cells into the human lung, with potential detrimental effects on the integrity of the lung tissue. Despite comprising the largest fractions of circulating lymphocytes in the lung, rather little is known about how peripheral blood natural killer (NK) cell and T cell subsets are equipped for lung-homing in COVID-19 and influenza. Here, we provide a detailed comparative analysis of NK cells and T cells in patients infected with SARS-CoV-2 or influenza virus, focusing on the protein and gene expression of chemokine receptors known to be involved in recruitment to the lung. For this, we used 28-colour flow cytometry as well as re-analysis of a publicly available single-cell RNA-seq dataset from bronchoalveolar lavage (BAL) fluid. Frequencies of NK cells and T cells expressing CXCR3, CXCR6, and CCR5 were altered in peripheral blood of COVID-19 and influenza patients, in line with increased transcript expression of *CXCR3*, *CXCR6*, and *CCR5* and their respective ligands in BAL fluid. NK cells and T cells expressing lung-homing receptors displayed stronger phenotypic signs of activation compared to cells lacking lung-homing receptors, and activation was overall stronger in influenza compared to COVID-19. Together, our results indicate a role for CXCR3^+^, CXCR6^+^, and/or CCR5^+^ NK cells and T cells that potentially migrate to the lungs in moderate COVID-19 and influenza patients, identifying common targets for future therapeutic interventions in respiratory viral infections.

## Introduction

The coronavirus disease 19 (COVID-19) pandemic, caused by the novel severe acute respiratory syndrome coronavirus 2 (SARS-CoV-2), and recurrent epidemics caused by influenza virus highlight the need for a better understanding of respiratory viral infections which have the potential to cause major global epidemics or pandemics. Future disease outbreaks with novel variants of these viruses affecting the airways are to be expected and prepared for.

Both SARS-CoV-2 and influenza share the same route of transmission and have highly overlapping symptoms and pathological features (1). During acute infection with respiratory viruses, the expression of specific chemokines mediating leukocyte recruitment are increased in the lung and bronchoalveolar lavage (BAL) fluid. These chemokines include CCL2, CCL3, CCL20, CXCL1, CXCL3, CXCL10, and IL8, attracting cells expressing chemokine receptors such as CCR2, CCR5, CXCR3, and CXCR6 (2–4). Patients suffering from severe COVID-19 exhibit exacerbated lung tissue damage likely resulting in a significant part from hyperactivated immune cells such as inflammatory macrophages (2), natural killer (NK) cells (5) and T cells (2, 6). Since lung-homing cytotoxic lymphocytes can contribute to lung pathology during acute infection, a better understanding of their major homing mechanisms will help in developing and improving treatment strategies in COVID-19, influenza, and presumably also other respiratory viral infections.

In this study, we investigated expression of lung-homing receptors and *in vivo* activation of NK cell and T cell subsets in the peripheral blood of patients suffering from moderate COVID-19 or influenza, and in healthy controls. The majority of patients infected with SARS-CoV-2 or influenza virus will experience mild or moderate disease. While moderate disease in COVID-19 and in influenza patients is per definition not fatal, patients may still require hospitalization and/or experience persistent long-term symptoms such as fatigue, respiratory problems, loss of taste or smell, headache, and diarrhea. An improved understanding of disease progression and the involvement of the immune system also in patients with moderate respiratory disease are important for better understanding the disease mechanisms and long-term development of therapeutic approaches. This study focuses on NK cells and T cells which belong to the first-line response to viral infections e.g. by cytotoxicity against infected cells and recruiting other cells to the site of infection. In addition to analyses by 28-colour flow cytometry, we analyzed gene expression in NK cells and T cells using a publicly available single-cell (sc)RNA-seq dataset of cells from bronchoalveolar lavage (7). Our data indicate a universal role for CXCR3-mediated lung-homing of NK cells and T cells in COVID-19 and influenza and an additional role for recruitment *via* CXCR6 and CCR5 in CD8^+^ T cells.

Together, we provide an extensive characterization of the lung-homing potential of NK cells and T cells in homeostasis and during acute respiratory viral infections with an emphasis on COVID-19 and influenza. The present results are of relevance for the understanding of the disease progression and for identifying target molecules to improve future therapeutic treatment strategies.

## Material and Methods

### Patients and Processing of Peripheral Blood

We enrolled a total of 10 hospitalized patients (four females and six males; age range 24-70; average age 55.3) who were diagnosed with COVID-19 by RT-qPCR for SARS-CoV-2 in respiratory samples. COVID-19 patients were sampled on average 11 days (range 6-16) after symptom onset. All of the COVID-19 patients were considered ‘moderate’ based on the guidelines for diagnosis and treatment of COVID-19 (Version 7) released by the National Health Commission and State Administration of Traditional Chinese Medicine (8). Furthermore, we enrolled 18 patients who tested positive for IAV (n=12) or IBV (n=6) (nine females and nine males; age range 21-84; median age 45) by RT-qPCR who were recruited during the four months immediately preceding the outbreak of COVID-19 in the Stockholm region. Influenza patients were sampled on average 4 days (range 1-11) after symptom onset. Nine of the 18 influenza patients were hospitalized.

None of the COVID-19 patients and only one of the influenza patients received immunosuppressive treatment. Diagnostics for all patients were performed at the diagnostic laboratory at the Karolinska University Hospital, Stockholm, Sweden. Mononuclear cells from peripheral blood were isolated by density gradient centrifugation (Lymphoprep). For each of the two separate cohorts, blood was collected from healthy blood donors and processed in parallel with patient samples. A detailed overview of the patient characteristics from all datasets used in this study is provided in **Table 1**.

**Table 1:** Clinical summary and demographics for patient cohorts included in study.

	Brownlie et al. (Protein)				Liao et al., 2020 (mRNA)	
*Sample type*	Peripheral blood				BAL fluid	
*Patient summary*	C-19 (*n* = 10)	Healthy (*n* = 21)	IAV/IBV (*n* = 18)	Healthy (*n* = 12)	C-19 (*n* = 9)	Healthy (*n* = 3)
Sampling (Days since symptom onset)	8 (3-13)	–	4 (1-10)*	–	13 (8-25)	–
Gender f/m	4/6	10/11	9/9	4/6	3/6	1/2
Age	55.3 (24-70)	47 (25-71)	45 (21-84)	45 (22-71)	51.9 (35-66)	28 (22-38)
Smoker	0 (0)	–	17 (3)*	–	–	–
*Pathology*	% (*n*)		% (*n*)		% (*n*)	
Bacterial pneumonia	0 (0)	–	11 (2)	–	–	–
Heart disease	0 (0)	–	22 (4)	–	–	–
Hypertension	0 (0)	–	24 (4)*	–	11 (1)	–
Chronic lung disease	0 (0)	–	11 (2)	–	–	–
Malignancy	0 (0)	–	17 (3)	–	–	–
**Symptoms**						
Fever	100 (10)	–	100 (17)*	–	100 (9)	–
Chest pain	40 (4)	–	61 (11)	–	22 (2)	–
Diarhoea	10 (1)	–	28 (5)	–	–	–
Myalgia	40 (4)	–	78 (14)	–	11 (1)	–
**Treatments**						
Oxygen treatment	100 (10) (2-9.5L)	–	38 (7) (0-4L)	–	–	–
Chest pain	40 (4)	–	61 (11)	–	22 (2)	–
Antibiotics	20 (2)	–	53 (8)**	–	–	–
Immunomodulation	0 (0)	–	6 (1)	–	11 (1)	–

Data points available *17/18, **15/18.

The study was approved by the Regional Ethical Review Board in Stockholm, Sweden, and by the Swedish Ethical Review Authority. All donors provided informed written consent prior to blood sampling.

### Transcriptome Analysis

Preprocessed and annotated scRNA-seq datasets of cells from BAL fluid from healthy controls and COVID-19 patients were obtained in RDS formats from published data (7). The data was read and analyzed using Seurat (4.0.5). Quality filtering and cell and cluster annotations were retained from the original analysis. The counts were scaled and transformed using the SCTransform function with mitochondrial gene expression regressed out before dimension reduction and clustering. For further visualization and analysis, the RNA counts were log normalized and scaled with mitochondrial reads regressed out, using the Seurat-implemented functions NormalizeData and ScaleData. Existing NK cells and T cell identities were confirmed by clustering and expression of canonical markers. CD4^+^ T cell, CD8^+^ T cell, and NK cell subsets were extracted and scaled before analysis of chemokine receptor and effector molecule expression based on patient groups. The code used for the scRNA-seq analysis can be found at https://github.com/Ingarod/BAL_Cov.

### Flow Cytometry

Antibodies and clones used for phenotyping are listed in **Supplementary Table 1**. Secondary staining was performed with streptavidin BB630 (BD Biosciences) and Live/Dead Aqua (Invitrogen). After surface staining, peripheral blood mononuclear cells (PBMC) were fixed and permeabilized using FoxP3/Transcription Factor staining kit (eBioscience).

Samples were analyzed on a BD LSR Symphony equipped with five lasers (355nm, 405nm, 488nm, 563nm, and 633nm) (BD Biosciences), and data were analyzed using FlowJo version 9.5.2 and version 10.7.1 (Tree Star Inc.).

### Statistical Analyses

GraphPad Prism 8 and 9 (GraphPad Software) was used for statistical analyses. The statistical method used is indicated in each figure legend.

## Results

### Altered Frequencies of NK Cell and T Cell Subsets Expressing Chemokine Receptors in Peripheral Blood in COVID-19 and Influenza Patients

**Figure 1:**
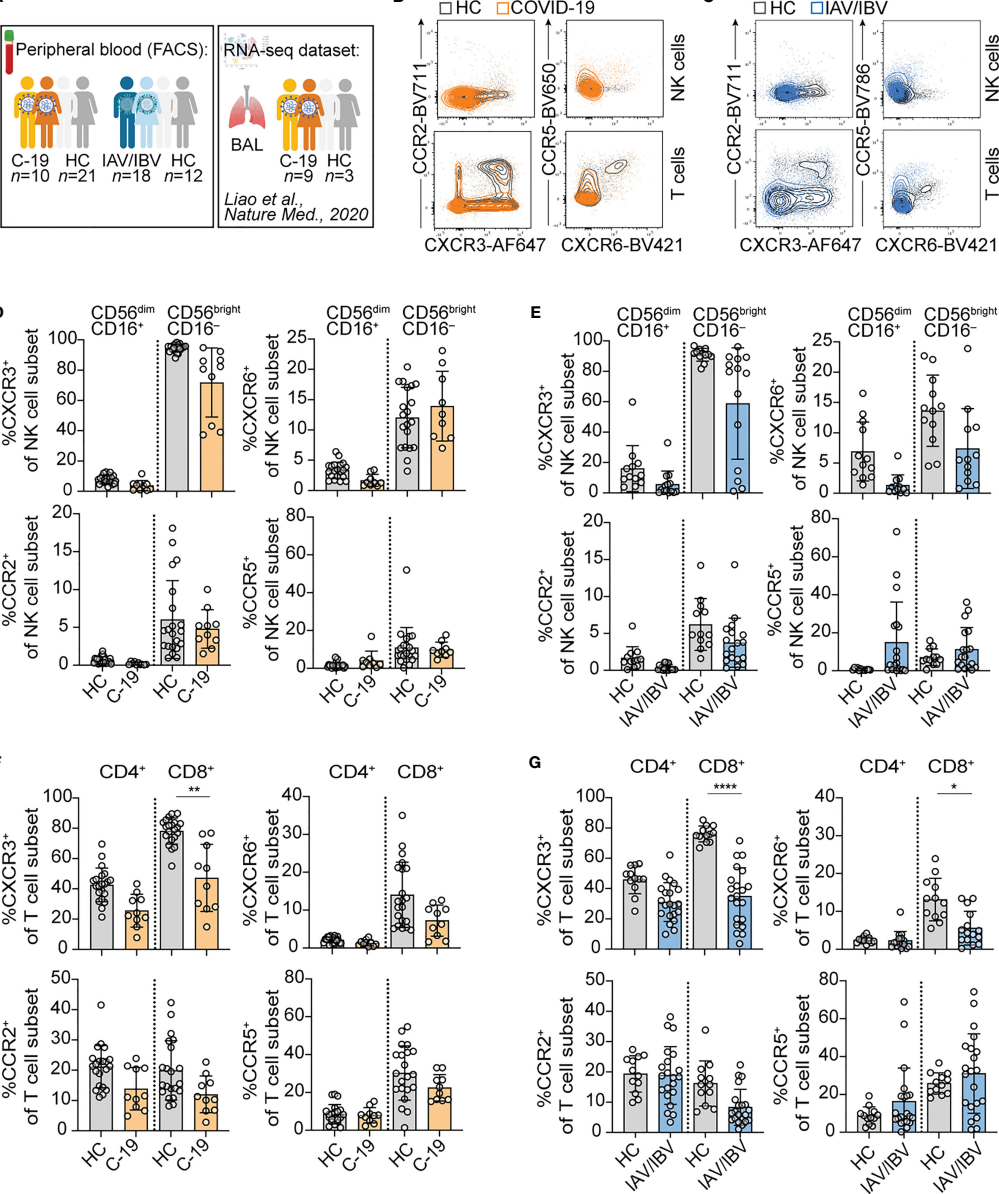
Altered composition of NK cells and T cells expressing different lung-homing receptors in peripheral blood in acute viral infection. **(A)** Schematic overview of study design. In brief, blood was collected from healthy controls, and from patients infected with SARS-CoV-2 or influenza A virus (IAV) or influenza B virus (IBV). Additionally, a publicly available RNA-seq dataset from BAL-fluid from COVID-19-infected patients published by Liao et al. (7) was re-analyzed. Figure created with BioRender.com **(B, C)** Representative overlays of NK cells (upper rows) and T cells (lower rows) of peripheral blood in a healthy control (HC) and a **(B)** COVID-19 patient or **(C)** an influenza patient, displaying expression of lung-homing receptors. **(D-G)** Summary of chemokine receptor expression on **(D, E)** CD56^dim^CD16^+^ and CD56^bright^CD16^-^ NK cells of **(D)** COVID-19 patients (C-19) or **(E)** influenza patients (IAV/IBV) (C-19: *n* = 10; IAV/IBV: CXCR3: *n* = 14; CXCR6: *n* = 12; CCR2: *n* = 18; CCR5: *n* = 18. HC: *n* = 12), or on **(F, G)** CD4^+^ and CD8^+^ T cells of **(F)** COVID-19-patients or **(G)** influenza patients (C-19: *n* = 10; IAV/IBV; CXCR3: *n* = 18; CXCR6: *n* = 13; CCR2: *n* = 18; CCR5: *n* = 18. HC: *n* = 12). **(D-G)** Wilcoxon matched-rank test. *p < 0.05, **p < 0.01, ****p < 0.0001.

To analyze lung-homing capacities of NK cells and T cells during acute respiratory viral infections, we first determined the expression of chemokine receptors relevant for lung-homing (CXCR3, CXCR6, CCR2, CCR5) on NK and T cell subsets in peripheral blood from COVID-19 and influenza patients as well as healthy controls (**Figure 1**; see **Figure S1A** for gating strategy).

In peripheral blood, both NK cells and T cells displayed an altered lung-homing receptor expression during acute infection with SARS-CoV-2 or influenza (**Figures 1B, C**). With respect to NK cells, all analyzed chemokine receptors were generally more frequently expressed on CD56^bright^CD16^–^ NK cells as compared to CD56^dim^CD16^+^ NK cells in both COVID-19 (**Figure 1D**) as well as influenza (**Figure 1D**). When compared to healthy controls, a trend towards a loss of CXCR3^+^ in COVID-19 and loss of CXCR3^+^ and CXCR6^+^ NK cells in influenza patients was observed (**Figures 1C, E**). In influenza patients, CCR5 expression was slightly increased on NK cells as compared to healthy controls (**Figure 1E**). In addition to the chemokine receptors predominantly expressed on CD56^bright^CD16^–^ NK cells, we found high expression of CXCR2 on peripheral blood CD56^dim^CD16^+^ NK cells both in healthy controls and COVID-19 patients (**Figures S2A, B**). The expression of CXCR2 was higher on CD56^dim^CD16^+^ NK cells lacking other lung-homing receptors (**Figure S2**). The frequency of CD56^dim^CD16^+^ NK cells expressing solely CXCR2 was significantly reduced in COVID-19 patients (**Figure S2D**).

Overall, T cells displayed similar expression patterns as NK cells in terms of lower frequencies of CXCR3^+^ CD8^+^ T cells in COVID-19 patients and of CXCR3^+^ and CXCR6^+^ CD8^+^ T cells in influenza patients (**Figures 1E, F**). Furthermore, there was a trend towards lower frequencies of CCR2^+^ T cells in COVID-19 and in influenza (**Figures 1F, G**). Except for CXCR3, chemokine receptor expression was generally higher on non-naïve T cell subsets, both in healthy controls and COVID-19 patients (**Figure S2G, H**; gating strategy in **Figure S1A**). CXCR3 was also highly expressed on naïve CD8^+^ T cells (**Figure S2H**) as reported before (9). CXCR2 was expressed mainly on CD8^+^ T cells, yet no differences between healthy and COVID-19 patients were observed (**Figure S2E, F**). For all chemokine receptors except CXCR2 a lower expression was observed on non-naïve T cells in COVID-19 patients (**Figure S2G, H**).

Loss of CXCR3 was observed in all non-naïve T cells in COVID-19 patients (**Figure S2G, H**), being significant in non-naïve CD8^+^ T cells as compared to healthy controls (**Figure S2H**). When comparing subsets of CD8^+^ memory T cells (TCM, TEM, TEMRA), patterns of lung-homing receptors differed between the subsets, with CXCR3 being highly expressed on all memory subsets and highest on CD4^+^ TEMRA (**Figure S2I**) and CD8^+^ TCM cells (**Figure S2J**). Loss of CXCR3 in COVID-19 patients was observed in all memory T cell subsets (**Figure S2I, J**). In contrast, CXCR6, CCR2, and CCR5 were most highly expressed on TEM cells both in healthy controls and COVID-19 patients (**Figure S2I, J**). Non-significant trends of reduction in CXCR6, CCR2, and CCR5 expression were observed in all memory T cell subsets (**Figure S2I, J**).

Together, the results indicate that the major subsets affected during moderate infection with COVID-19 or influenza are lymphocytes expressing CXCR3 as well as CXCR6 and CCR5. Direct comparative analyses in COVID-19 and influenza patients suggest common lung-homing capacities but also potential differences between NK cell- and T cell-lung homing in the two diseases.

### Common Activation Profiles in Peripheral Blood NK Cells Expressing Lung-Homing Receptors in COVID-19 and Influenza Patients

**Figure 2:**
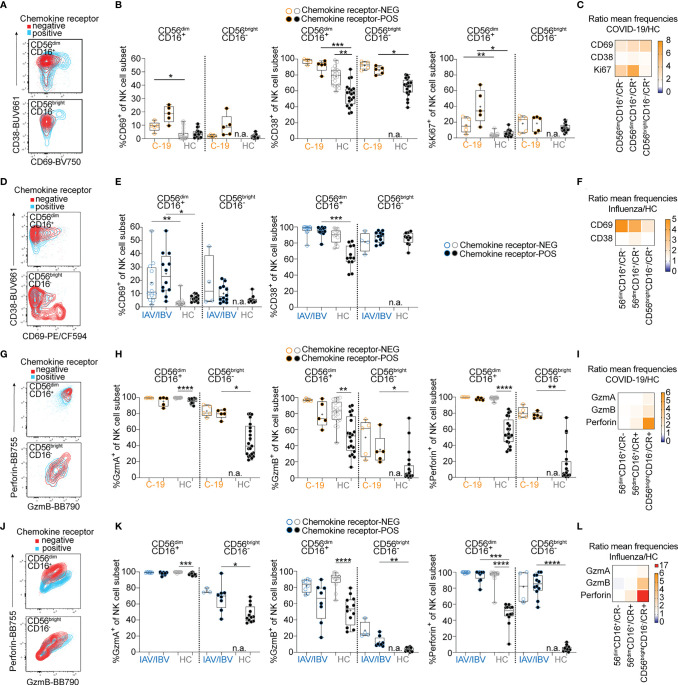
Lung-homing receptor-positive NK cells display an activated phenotype in peripheral blood of COVID-19 and influenza patients. Chemokine receptor-positive cells in COVID-19 and influenza patients were identified using the Boolean gate “CXCR3^+^ OR CXCR6^+^ OR CCR2^+^ OR CCR5^+^” (‘CR+’; see representative gates in **Figure S1B**). Cells lacking all of these receptors were identified as chemokine receptor-negative (CR–). **(A)** Representative overlays and **(B)** summary of data showing CD69, CD38 and Ki67 expression on chemokine receptor-negative (open circles) and chemokine receptor-positive (closed circles) CD56^dim^CD16^+^ and CD56^bright^CD16^-^ blood NK cells from COVID-19 patients (orange, *n* = 5-10) and healthy controls (grey, *n* = 20). **(C, F)** Heatmaps displaying the ratio of mean expression of **(C)** CD69, CD38, and Ki67 between COVID-19 patients and healthy controls or **(F)** of CD69 and CD38 between influenza patients and healthy controls in CR^-^ and CR^+^ CD56^dim^CD16^+^ and CR^+^ CD56^bright^CD16- NK cells. Baseline value = 1 (white). **(D)** Representative overlays and **(E)** summary of data of CD69 and CD38 expression on CR^+^ and CR^–^ CD56^dim^CD16^+^ and CD56^bright^CD16^-^ blood NK cells from influenza patients (*n* = 4-12) and healthy controls (grey, *n* = 12), respectively. **(G)** representative dot plots and **(H)** summary of data showing granzyme A, granzyme B and perforin expression on chemokine receptor-negative and -positive CD56^dim^CD16^+^ and CD56^bright^CD16^-^ blood NK cells from COVID-19 patients (*n* = 5-10) and healthy controls (*n* = 20). **(I, L)** Heatmaps displaying the ratio of mean expression of **(I)** granzyme A, granzyme B and perforin between COVID-19 patients and healthy controls or **(L)** between influenza patients and healthy controls in CR^-^ and CR^+^ CD56^dim^CD16^+^ and CR^+^ CD56^bright^CD16- NK cells. Baseline value = 1 (white). **(J)** Representative overlays and **(K)** summary of data of granzyme A, granzyme B, and perforin expression on chemokine receptor-negative and -positive CD56^dim^CD16^+^ and CD56^bright^CD16^-^ blood NK cells from influenza patients (*n* = 4-12) and healthy controls (*n* = 12), respectively. **(B, E, H, K)** Box and Whiskers, min to max, mean shown as ‘+’. Kruskal-Wallis rank-sum test with Dunn’s *post hoc* test for multiple comparisons. *p < 0.05, **p < 0.01, ***p < 0.001, ****p < 0.0001.

**Figure 3:**
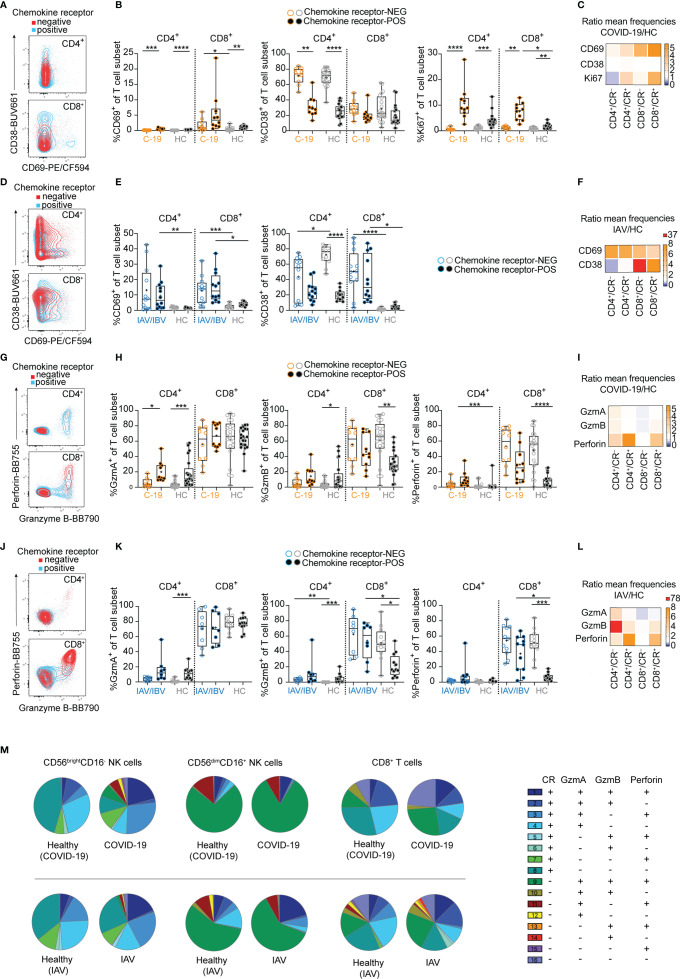
Lung-homing receptor-positive T cells are activated *in vivo* in COVID-19 and influenza. Chemokine receptor-positive (CR+) cells in COVID-19 and influenza patients were identified using the Boolean gate “CXCR3^+^ OR CXCR6^+^ OR CCR2^+^ OR CCR5^+^” (see representative gates in **Figure S1B**). Cells lacking all of these receptors were identified as chemokine receptor-negative (CR–). **(A)** Representative overlays and **(B)** summary of data showing CD69, CD38 and Ki67 expression on chemokine receptor-negative and -positive CD4^+^ and CD8^+^ blood T cells from COVID-19 patients (*n* = 10) and healthy controls (*n* = 21). **(C, F)** Heatmaps displaying the ratio of mean expression of **(C)** CD69, CD38 and Ki67 between COVID-19 patients and healthy controls or **(F)** of CD69 and CD38 between influenza patients and healthy controls in CR^-^ and CR^+^ CD4^+^ and CD8^+^ blood T cells. Baseline value = 1 (white). **(D)** Representative overlays and **(E)** summary of data of CD69 and CD38 expression on chemokine receptor-negative and -positive CD4^+^ and CD8^+^ blood T cells from influenza patients (*n* = 4-12) and healthy controls (*n* = 12), respectively. **(G)** representative overlays and **(H)** summary of data showing granzyme A, granzyme B and perforin expression on chemokine receptor-negative and -positive CD4^+^ and CD8^+^ blood T cells from COVID-19 patients (*n* = 10) and healthy controls (*n* = 20). **(I, L)** Heatmaps displaying the ratio of mean expression of **(I)** granzyme A, granzyme B and perforin between COVID-19 patients and healthy controls or **(L)** between influenza patients and healthy controls in CR^-^ and CR^+^ CD4^+^ and CD8^+^ blood T cells. Baseline value = 1 (white). **(J)** Representative overlays and **(K)** summary of data of granzyme A, granzyme B, and perforin expression on chemokine receptor-negative and -positive CD4^+^ and CD8^+^ blood T cells from influenza patients (*n* = 8-11) and healthy controls (*n* = 12), respectively. **(M)** SPICE analysis of CD56^bright^CD16^-^ NK cells (left), CD56^dim^CD16^+^ NK cells (middle), and CD8^+^ T cells (right) in COVID-19 patients (upper row) and influenza patients (lower row) and the respective healthy controls, displaying co-expression of effector molecules in CR^-^ and CR^+^ cells. *n* = 10/21 (COVID-19/healthy), *n* = 6/12 (influenza/healthy). **(B, E, H, K)** Box and Whiskers, min to max, mean shown as ‘+’. Kruskal-Wallis rank-sum test with Dunn’s *post hoc* test for multiple comparisons. *p < 0.05, **p < 0.01, **p < 0.001, ****p < 0.0001.

We and others have previously demonstrated an activated phenotype in peripheral blood NK cells and T cells in COVID-19 and influenza, respectively (5, 10, 11). Here, we aimed to determine the expression of NK and T cell activation markers in relation to expression of lung-homing receptors. For this purpose, we identified chemokine receptor-positive cells in COVID-19 and influenza patients by boolean gating, combining cells expressing CXCR3, CXCR6, CCR2, and/or CCR5 (**Figures 2**, **3**, See gating strategy in **Figure S1B, C**). In COVID-19 patients, NK cells expressing these lung-homing receptors displayed higher expression of CD69 (on CD56^bright^CD16^–^ and CD56^dim^CD16^+^ NK cells) and Ki67 (in CD56^dim^CD16^+^), as compared to lung-homing receptor-negative NK cells (**Figures 2A–C**). Expression of CD38 was overall high but slightly lower on lung-homing receptor-positive NK cells (**Figure 2B**). Due to very low numbers of CD56^bright^CD16^–^ NK cells lacking any relevant chemokine receptor in healthy controls, no comparisons could be performed for chemokine receptor-negative CD56^bright^CD16^-^ NK cells.

In influenza patients, the frequency of CD69^+^ NK cells was slightly higher in lung-homing receptor-negative NK cells (**Figures 2D–F**), but overall higher than in COVID-19 patients. The frequency of CD38^+^ NK cells was similar in COVID-19 and influenza patients (**Figures 2C, F**). These data indicate general differences in activation patterns for NK cells in COVID-19 and influenza patients. Expression of effector molecules (granzymes, perforin) was increased in NK cells in both COVID-19 (**Figure 2G–I** and **Figure S3A, C, D**) and influenza patients (**Figures 2J–L** and **Figure S3B, E, F**), both in frequency of positive NK cells and in expression levels. Significant increases in expression levels of granzyme A, granzyme B, and perforin were particularly observed in CD56^bright^CD16^-^ NK cells (**Figure S3D, F**). In healthy controls as well as COVID-19 and influenza patients, overall effector molecule expression was highest in lung-homing receptor-negative NK cells (**Figures 2G, H, J, K**). However, the expression of granzymes and perforin was strongest in CD56^bright^CD16^-^ NK cells expressing lung-homing receptors (**Figures 2H, I, K, L**). Relative to healthy controls, influenza patients had higher perforin upregulation than COVID-19 patients (**Figures 2I, L**), indicating stronger activation of peripheral blood NK cells in influenza.

Together, these results demonstrate common activation patterns between NK cell subsets in COVID-19 and influenza patients, with a stronger activation of NK cells expressing CXCR3, CXCR6, CCR2, and/or CCR5 than NK cells lacking any lung-homing receptors. Furthermore, blood NK cells in influenza patients generally displayed stronger signs of activation compared to COVID-19 patients with moderate disease.

### Different Activation Patterns Between COVID-19 and Influenza in NK Cells and T Cells

Since both NK cells and T cells displayed a loss of lung-homing receptor-positive cells in COVID-19 and influenza patients (**Figure 1**), we next sought to determine whether activation patterns differed between lung-homing receptor-positive and -negative T cells (**Figure 3**). In COVID-19 patients CD69 expression was biased towards CD8^+^ T cells co-expressing lung-homing receptors (**Figures 3A–C**). In contrast, CD69 upregulation was overall higher and more uniform between the T cell subsets in influenza (**Figures 3D–F**). Furthermore, CD38 was strongly upregulated on CD8^+^ T cells in influenza but not COVID-19 patients (**Figures 3B–F**). Finally, expression of Ki67 was largely confined to chemokine receptor-positive T cells, both in healthy controls and in COVID-19 patients (**Figures 3B, C**). Upregulation of Ki67 was strongest in lung-homing receptor-positive CD8^+^ T cells (**Figure 3C**), which is in line with patterns observed in CD56^dim^CD16^+^ chemokine receptor-positive NK cells (**Figure 2C**), indicating a particular activation of cytotoxic lymphocytes expressing lung-homing receptors in COVID-19 patients.

In comparison to NK cells where upregulation of effector molecules was more uniform between COVID-19 and influenza patients (**Figure 2**), differences were more distinct for T cells (**Figures 3G–L**). As expected, expression of perforin and granzymes was to a large extent contained to cytotoxic CD8^+^ T cells (**Figures 3G, H, J, K**), although some expression was also observed in lung-homing receptor-positive CD4^+^ T cells in COVID-19 and influenza patients (**Figures 3G, H, J, K**). Expression of granzyme B and perforin was highest in lung-homing receptor-negative CD8^+^ T cells in healthy controls as well as in COVID-19 and influenza patients (**Figures 3H, K**). Importantly however, a significant increase in effector molecule expression was mainly found in the lung-homing receptor-positive CD8^+^ T cell subset (**Figures 3K, L**).

Stratification of co-expression of lung-homing receptors and specific effector molecules showed some variation but also similarities between all cytotoxic cell subsets (CD56^bright^CD16^-^/CD56^dim^CD16^+^ NK cells and CD8^+^ T cells) and between COVID-19 and influenza (**Figure 3M**). In detail, the frequency of GzmA^+^GzmB^+^perforin^+^ cells co-expressing lung-homing receptors was commonly strongly increased in patients, except for CD56^dim^CD16^+^ NK cells in COVID-19 patients. This increase in cells co-expressing multiple effector molecules was accompanied with a specific reduction of GzmA^+^GzmB^-^perforin^-^ lung-homing receptor-positive cells. Despite this increase in effector molecule expression and while almost all NK cells expressed effector molecules, the frequency of CD8^+^ T cells lacking both effector molecules and lung-homing receptors increased in COVID-19 patients. No or only minor changes were seen for GzmA-single-positive NK cells and T cells lacking lung-homing receptors (**Figure 3M**).

Finally, direct comparison of effector molecule expression levels between NK cells and T cells as well as between COVID-19 and influenza patients revealed similarities but also slightly different patterns between the two diseases: Granzyme A and B were significantly increased in CD56^bright^CD16^-^ NK cells and CD4^+^ T cells in COVID-19 patients (**Figure S3D**), while granzyme B and perforin levels were significantly higher in NK cells and T cells in influenza patients (**Figure S3F**).

Together, these data indicate an overall stronger *in vivo* priming of T and NK cells co-expressing lung-homing receptors as compared to their counterpart lacking lung-homing receptors. Furthermore, upregulation of activation markers on T cells and NK cells was stronger in influenza patients as compared to COVID-19 patients. Hence, lymphocytes with a capacity of cytotoxic function display a broader pattern of activation in influenza patients as compared to COVID-19 patients with moderate disease.

### Accumulation of Phenotypically Armed NK Cells and T Cells Expressing Lung-Homing Receptors in BAL Fluid of COVID-19 Patients

The loss of NK cells and T cells expressing lung-homing receptors in the peripheral blood of COVID-19 and influenza patients suggests migration of the respective cells to the infected lung tissue. This would lead to an accumulation of NK cells and T cells in the lung with different characteristics as compared to healthy controls.

**Figure 4:**
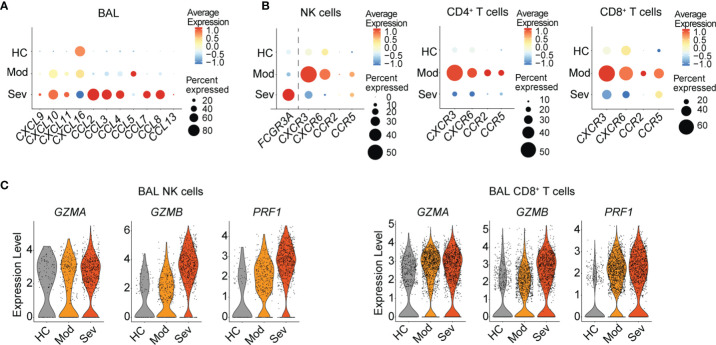
Gene expression of chemokines, chemokine receptors, and effector molecules in NK cells and T cells in BAL fluid in patients with moderate and severe COVID-19. **(A)** Dot plots of average chemokine RNA expression in total BAL fluid cells. **(B)** Average gene expression and proportion of *FCGR3A* and chemokine receptors in NK cells as well as chemokine receptors in CD4^+^ and CD8^+^ T cells from BAL fluid from healthy controls (*n* = 3) and COVID-19 patients with moderate (*n* = 3) or severe (*n* = 6) disease. **(C)** Violin plots of RNA expression of effector molecules in BAL fluid NK cells and in CD8^+^ T cells in healthy controls and moderate and severe COVID-19 patients. **(A–C)** The respective scRNA-seq dataset is derived from Liao et al. (7).

In order to analyze changes in NK cells and T cells in the lung upon respiratory viral infection, we analyzed gene expression of chemokines that are ligands to CXCR3, CXCR6, CCR2, and CCR5 (**Figure 4A**) or total chemokines (**Figure S4A**) in total BAL cells as well as chemokine receptors in NK cells and CD8^+^ T cells (**Figure 4B** and **Figure S4B**) using a publicly available scRNAseq dataset from BAL fluid cells from COVID-19 patients with moderate or severe disease (7). High transcript levels of a large number of chemokines were found in patients with severe disease (**Figure 4A** and **Figure S4A**). In comparison, in patients with moderate disease, increases in transcript levels were limited to *CXCL9*/*CXCL10*/*CXCL11*, *CXCL16*, and *CCL5*, which are encoding ligands for CXCR3, CXCR6, and CCR5, respectively. In line with these results, transcripts for *CXCR3*, *CXCR6*, and *CCR5* were highly enriched in NK cells as well as CD4^+^ and CD8^+^ T cells in COVID-19 patients with moderate disease, suggesting a highly specific accumulation of cells expressing the respective receptors in the lung (**Figure 4B**). In patients with severe disease, gene expression of chemokine receptors was low as compared to patients with moderate disease and even healthy controls (**Figure 4B**). High expression of *FCGR3A*, encoding CD16 indicated a high frequency of CD56^dim^CD16^+^ NK cells in BAL fluid in patients with severe disease (**Figure 4B**). CD56^dim^CD16^+^ NK cells largely lack expression of the chemokine receptors analyzed in this study, explaining the corresponding low gene expression of chemokine receptors in these patients. Although it is possible that some of the cells in BAL fluid of patients with moderate disease are comprised of tissue-resident NK cells and memory T cells which express high levels of CXCR3 and CXCR6 at the transcriptional and protein levels (12, 13), our data strongly suggest specific infiltration of NK cells and T cells from peripheral blood into the lung in COVID-19 patients with moderate disease. Other chemokines potentially important for NK and T cell recruitment in patients with moderate disease are CCL18 and HMGB1, due to their increased expression in BAL fluid of these donors (**Figure S4A**). While the putative cognate receptor for CCL18, *CCR8*, is not expressed on BAL NK cells and T cells (**Figure S4B**), *CXCR4*, the cognate receptor for CXCL12 is highly expressed on BAL NK cells and CD4^+^ T cells (**Figure S4B**). CXCL12 is forming a complex with HMGB1 which enables signaling through CXCR4 (14). *CXCR4* expression was overall low in BAL NK and T cells from patients with moderate disease but high in patients with severe disease (**Figure S4B**). Hence, further investigations are needed for understanding CXCR4-mediated lymphocyte recruitment to the lung in respiratory viral infections.

Since NK cells and CD8^+^ T cells from peripheral blood displayed upregulated levels of effector molecules, these cells might have important cytotoxic implications upon infiltration into the lung. Indeed, NK cells and CD8^+^ T cells in BAL fluid from COVID-19 patients displayed increased transcript expression levels of *GZMA*, *GZMB*, and *PRF1* even in patients with moderate disease (**Figure 4C**).

Altogether, our data suggest distinct recruitment of activated CXCR3^+^ and CXCR6^+^ NK cells and T cells to the lung in patients with moderate COVID-19 and influenza, indicating overlapping recruitment mechanisms in these two respiratory viral infections despite minor differences in effector molecule expression. A better understanding of lung-homing of innate and adaptive cytotoxic lymphocytes in patients with respiratory viral infections might reveal universal concepts of disease progression in these two, and possibly other, respiratory viral infections.

## Discussion

The COVID-19 pandemic has raised awareness about the need for a better understanding of the course of respiratory viral infections. So far, only a few studies have compared cellular immune responses in COVID-19 and other respiratory viral infections side by side (15–17). Both SARS-CoV-2 and influenza cause respiratory disease with similar disease presentation ranging from asymptomatic or mild to severe disease and death. Despite parallels between COVID-19 and influenza, both diseases differ at an immunological level, for example, SARS-CoV-2 does not infect NK cells, T cells, or other mononuclear blood cells due to the lack of ACE2 (18), while influenza virus has been suggested to infect NK cells (19). Furthermore, NK cells can directly recognize the viral protein Hemagglutinin (HA) of influenza virus by NKp46 (20), while SARS-CoV-2 S protein has been suggested to bind to NKG2D (21), suggesting differences in virus-dependent mechanisms of NK cell activation. In contrast to influenza virus, SARS-CoV-2 can spread to other organs if not cleared efficiently from the respiratory tract (22), and the viruses induce different antiviral responses in lung epithelial cells (23). Therefore, patterns of leukocyte activation and trafficking in respiratory viral infections are of interest for identifying and targeting common molecular pathways for therapeutic interventions (24). While a broad perturbation of the immune system and a distinct recruitment of myeloid cells to the lung has been shown for patients with severe COVID-19 (25), the regulation of lymphocyte recruitment to the lung has been investigated less, in particular in patients with moderate disease.

Here, we compared changes in NK cell and T cell subset compositions, focusing on expression of the lung-homing receptors CXCR3, CXCR6, CCR2, and CCR5, in the peripheral blood from patients with clinically moderate COVID-19 or influenza. Peripheral blood NK cells and T cells, in particular CD8^+^ T cells, largely overlapped in their lung-homing receptor expression profile in COVID-19 and influenza patients. This indicates similar lung-homing mechanisms for cytotoxic lymphocytes in both diseases. We identified a stronger loss of CXCR3^+^ and CXCR6^+^ CD8^+^ T cells and overall stronger activation of NK cells and T cells in the blood of influenza patients as compared to COVID-19 patients, in line with the recent observation of a lower inflammatory profile in COVID-19 patients as compared to influenza patients (15). The activated profile of NK cells and CD8^+^ T cells, including elevated expression of CD69 and Ki67, and induction of perforin, is biased towards subsets co-expressing lung-homing receptors. Levels of corresponding leukocyte-recruiting chemokines such as CXCL10, CCL5, and CXCL5 are elevated in the BAL fluid and in nasopharyngeal swabs of patients infected with SARS-CoV-2 (26–28), supporting our hypothesis of the importance of these chemokines for recruitment of lymphocytes expressing CXCR3, CCR5, and potentially also CXCR2. Monocytes producing ligands for CXCR3 have been shown to be expanded in the lungs of COVID-19 patients (7), and although not significant, CXCL10 mRNA expression was increased in monocytic macrophages from BAL fluid from COVID-19 patients with moderate disease (2). This may contribute to elevated lung infiltration of CXCR3^+^ lymphocytes in COVID-19 patients. In contrast, data on chemokine expression in the lung of influenza-virus infected patients are rather limited. CXCL10 (IP-10) is strongly increased in BAL fluid (29) and lung tissue (3) of severe or fatal cases, respectively, but not increased in BAL fluid of patients with moderate disease (29). Interestingly, cytokine and chemokine production were higher in severe influenza as compared to severe COVID-19 (29), but more data on the direct comparison between COVID-19 and influenza patients at moderate level are needed. In serum, CXCL10 has been shown to be upregulated in influenza patients (30, 31) and in SARS patients patients with ARDS (32). Changes in chemokine levels in peripheral blood of COVID-19 and influenza patients have been addressed by multiple studies, in particular CXCL9, CXCL10, CXCL11, CXCL16, CCL2, and CCL5 were altered in patients as compared to controls, and levels correlated to disease severity (23, 27, 33–36). Finally, CXCR3^+^ cells are overall increased in the lung of Rhesus macaques following infection with SARS-CoV-2 (37), and CXCL10 levels were increased in *in vitro* influenza virus-infected human macrophages (38), in human lung tissue explants infected with SARS-CoV-2 (26), and in the lungs of mice infected with influenza virus (4, 39, 40).

Together, this suggests a predominant role for the CXCR3:CXCL10 axis for lung-homing upon respiratory viral infection and also in non-viral lung tissue injury (41). Elevated CXCL10 levels in BAL were associated with longer duration of mechanical ventilation in COVID-19 patients (27). In mice, CXCR3-deficiency rescued CCR5-deficient mice from IAV-induced mortality (42). Other murine IAV infection models demonstrated a role for CXCR3 and CCR5 for NK cell lung-homing and showed NK cell accumulation in the lung was not due to proliferation or apoptosis (40). For CD8^+^ T cells, murine models demonstrated that virus-specific T cells express CXCR3 and migrate to CXCR3 ligands *in vitro* (43). Furthermore, CCR5 is required for recruitment of memory CD8^+^ T cells to IAV-infected epithelium and is rapidly upregulated on the surface of memory CD8^+^ T cells upon viral challenge (43). Interestingly, these mouse models also revealed that CCR5 is required for circulating CD8^+^ memory T cells to migrate to respiratory airways but not lung parenchyma during virus challenge (43), indicating potential distinct migration patterns depending on chemokine receptor expression. In addition to CCR5 and CXCR3, CXCR6 has been suggested to be of importance for recruitment of resident memory T cells to the airways both in mice (13) and in patients with moderate COVID-19 (7). Indeed, our analysis of RNA-seq data from BAL fluid of COVID-19 patients suggest a CXCR3- and CXCR6-mediated recruitment of NK cells and T cells, as well as a CCR5-mediated recruitment particularly of CD8^+^ T cells in moderate disease. In severe disease, myeloid cells which likely derive from circulation, comprise the major subset infiltrating the lung (25). In these patients, hyperinflammation will lead to lung tissue damage, accompanied with vascular permeability, pulmonary capillary leakage (44, 45) and oedema with an increased extravascular lung water index (46). Hence, infiltration of NK cells and T cells might be rather unspecific during severe disease due to capillary leakage. This is supported by a high expression of FCGR3A, encoding CD16, in the BAL fluid NK cells of patients with severe COVID-19 as it indicates a high frequency of CD56^dim^CD16^+^ NK cells. These cells constitute the majority of NK cells in the circulation and largely lack expression of the chemokine receptors analyzed in this study, explaining the overall low chemokine receptor gene expression in patients with severe COVID-19. In patients with severe COVID-19, CXCR4 might play a crucial role in recruitment of NK cells and T cells as indicated by our and previous analysis of the BAL fluid dataset (47), but further studies are needed to unravel CXCR4-mediated lung-infiltration.

The relevance of the CXCR3:CXCL10 axis for lung tissue-homing of cytotoxic immune cells such as NK cells and CD8^+^ T cells might be of interest for future approaches of intervention. In critical patients with severe COVID-19, pharmacologic inhibition of e.g. the CCR1 and/or CCR5 (2, 48) or the CXCL10:CXCR3 and the CXCL16:CXCR6 pathways (49, 50), respectively, were suggested to suppress hyperinflammation. Despite not being life-threatening, moderate respiratory infection can require hospitalization, can progress into severe disease, and/or can result in long-term consequences such as ‘long COVID’. Given that hyperactivated cytotoxic lymphocytes including NK cells and CD8^+^ T cells might play an important role in tissue injury, a targeted therapeutic approach appears desirable also in patients with moderate respiratory disease. Based on our results, we support focusing on CXCL10:CXCR3 and CXCL16:CXCR6 also in moderate disease as an early intervention for mitigating short- and long-term complications in these patients. This approach might be globally relevant for respiratory viral infections since e.g. antibody-mediated targeting of CXCL10 also improved survival of H1N1-infected mice (51). Furthermore, the role of circulating NK cells and T cells in COVID-19 and in influenza in potentially facilitating the recruitment of other cell types such as neutrophils (24) or other T cells (47) needs to be considered, and the potential immunoregulatory roles of NK cells and T cells in the human lung as well as other organs in health and viral disease remains to be studied further (52).

While our study benefits from access to cells from peripheral blood as well as to a RNA-seq dataset from BAL fluid of COVID-19 patients with moderate disease, a shortcoming of our study is a low number of patients, impeding a detailed stratification by clinical or other parameters, such as age, gender, and comorbidities. Another limitation is the indirect comparison of BAL fluid to protein analyses since the cohorts were not identical, and BAL fluid was not available from our cohort. Analysis of chemokines at the local site of infection in correlation to the chemokine receptor expression of lymphocytes in blood and lung would be highly relevant for this study. However, we believe that the publicly available RNA-seq data offers great value in complementing our study. Furthermore, when directly comparing COVID-19 and influenza, the differences in time-course and pathophysiology of the two viral diseases need to be considered. A longer interval between infection and onset of symptoms in COVID-19 patients as compared to the rapid onset of disease in influenza patients might result in differences in NK cell and T cell activation and phenotypic patterns, and large multicenter studies are required to validate our and other groups’ findings before they can be applied in a clinical setting. Finally, future studies will assess how acute respiratory viral infection with SARS-CoV-2, influenza viruses, and other viruses affects the landscape of activated NK cells and T cells in the lung.

Together, our results strongly implicate the importance of, in particular, CXCR3 as a lung-homing receptor in respiratory viral infections such as SARS-CoV-2 and influenza virus in humans. The results also reveal a role for other receptors such as CXCR6 and CCR5 on CD56^bright^CD16^–^ NK cells and CD8^+^ T cells as potential alternative receptors of importance. A better understanding of how these chemokine receptors affect disease progression might help to develop future immunotherapeutic interventions in patients that developed disease in current or future epidemics or pandemics with respiratory viral infections.

## Data Availability Statement

The raw data supporting the conclusions of this article will be made available by the authors, without undue reservation.

## Ethics Statement

The studies involving human participants were reviewed and approved by the Regional Ethical Review Board in Stockholm, Sweden, Swedish Ethical Review Authority, Stockholm, Sweden. The patients/participants provided their written informed consent to participate in this study.

## Author Contributions

Conceptualization/study design: DB, MB, SG-R, AS-S, and NM. Investigation: DB, IR, RV, SF-J, SV, JM, and NM. Resources: HA, HG, SF-J, SV, SG-R, and AS-S. Writing – original draft: DB and NM. Writing – review and editing: DB, IR, RV, HA, HG, SF-J, SV, MB, H-GL, JM, SG-R, AS-S, and NM. All authors contributed to the article and approved the submitted version.

## Funding

This work was supported by the Swedish Research Council (NM: 2021-01039, AS-S: 2020-00896, 2020-05764, 2020-06100, 2020-06312, 2021-03046), the Center for Innovative Medicine (CIMED, NM: 20200680, SR: 20180850, 20200710), the Groschinsky Foundation (NM: M2017), the Åke Wibergs Foundation (NM: M18-0183), the Karolinska Institutet Foundation (NM: 2018-01663, AS-S: 2020-01397) and the Tornspiran Foundation (NM), Nordstjernan AB (H-GL), and the Knut and Alice Wallenberg Foundation (H-GL), the Bill and Melinda Gates Foundation (AS-S: 4-2360/2020). SG was supported by Region Stockholm (clinical research appointment and ALF project).

## Conflict of Interest

The authors declare that the research was conducted in the absence of any commercial or financial relationships that could be construed as a potential conflict of interest.

## Publisher’s Note

All claims expressed in this article are solely those of the authors and do not necessarily represent those of their affiliated organizations, or those of the publisher, the editors and the reviewers. Any product that may be evaluated in this article, or claim that may be made by its manufacturer, is not guaranteed or endorsed by the publisher.
