# Supplementary figures and images for: Comparison of Lung-Homing Receptor Expression and Activation Profiles on NK Cell and T Cell Subsets in COVID-19 and Influenza
Source: Front Immunol. 2022 Mar 16;13:834862. doi: 10.3389/fimmu.2022.834862 (PMC8966396; doi:10.3389/fimmu.2022.834862)

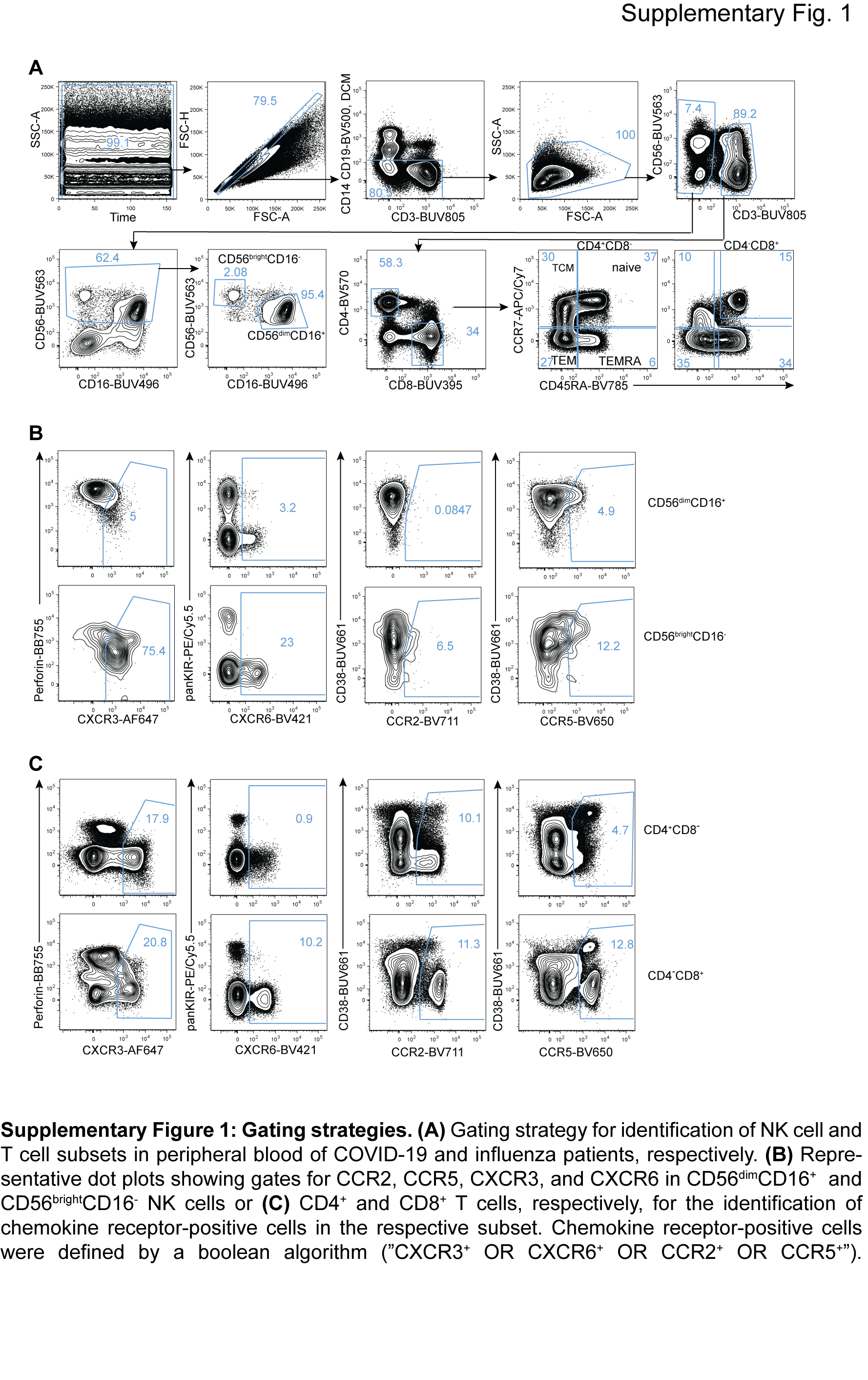

Supplement: Supplementary file 1 [file Image_1.tif]

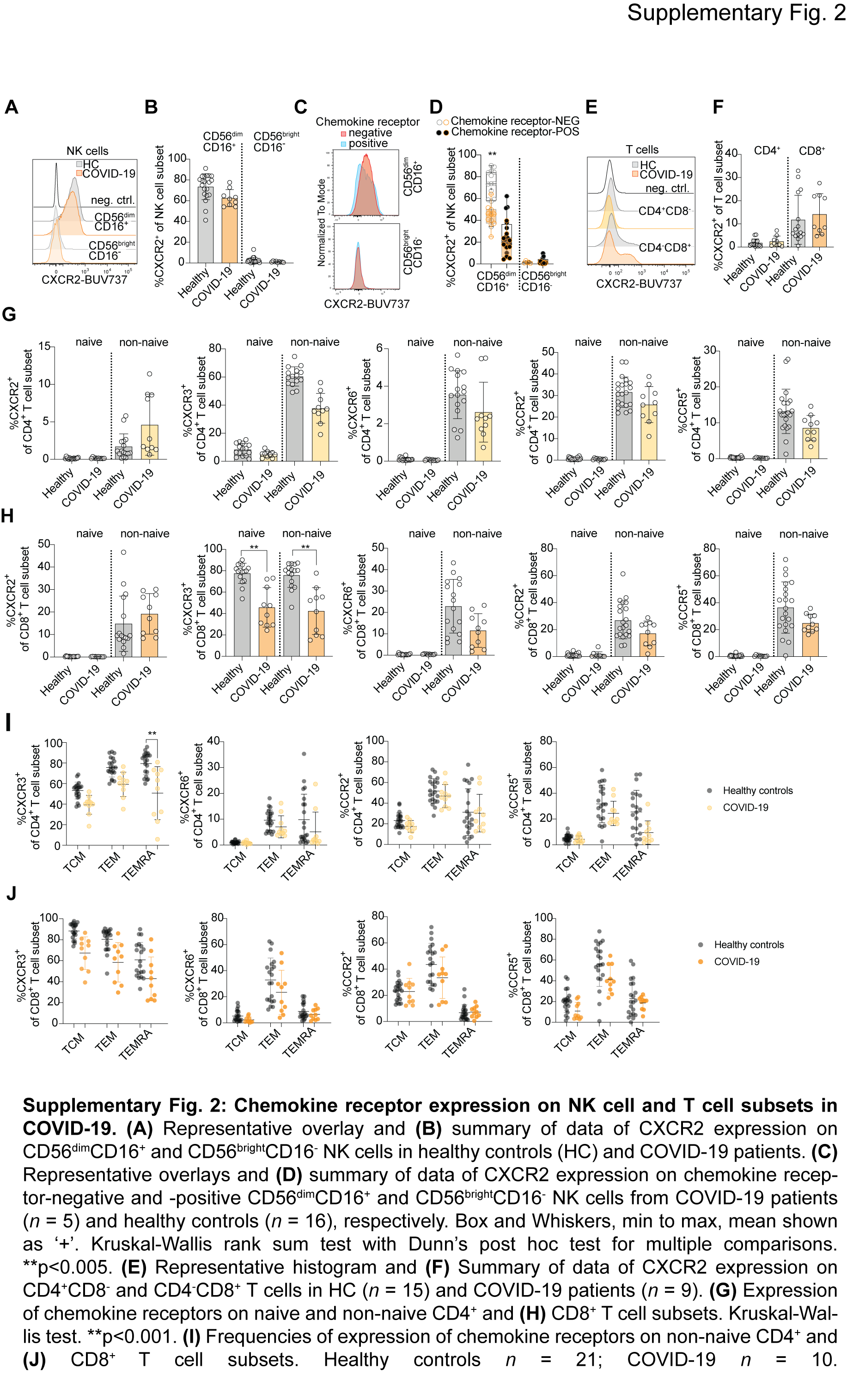

Supplement: Supplementary file 2 [file Image_2.tif]

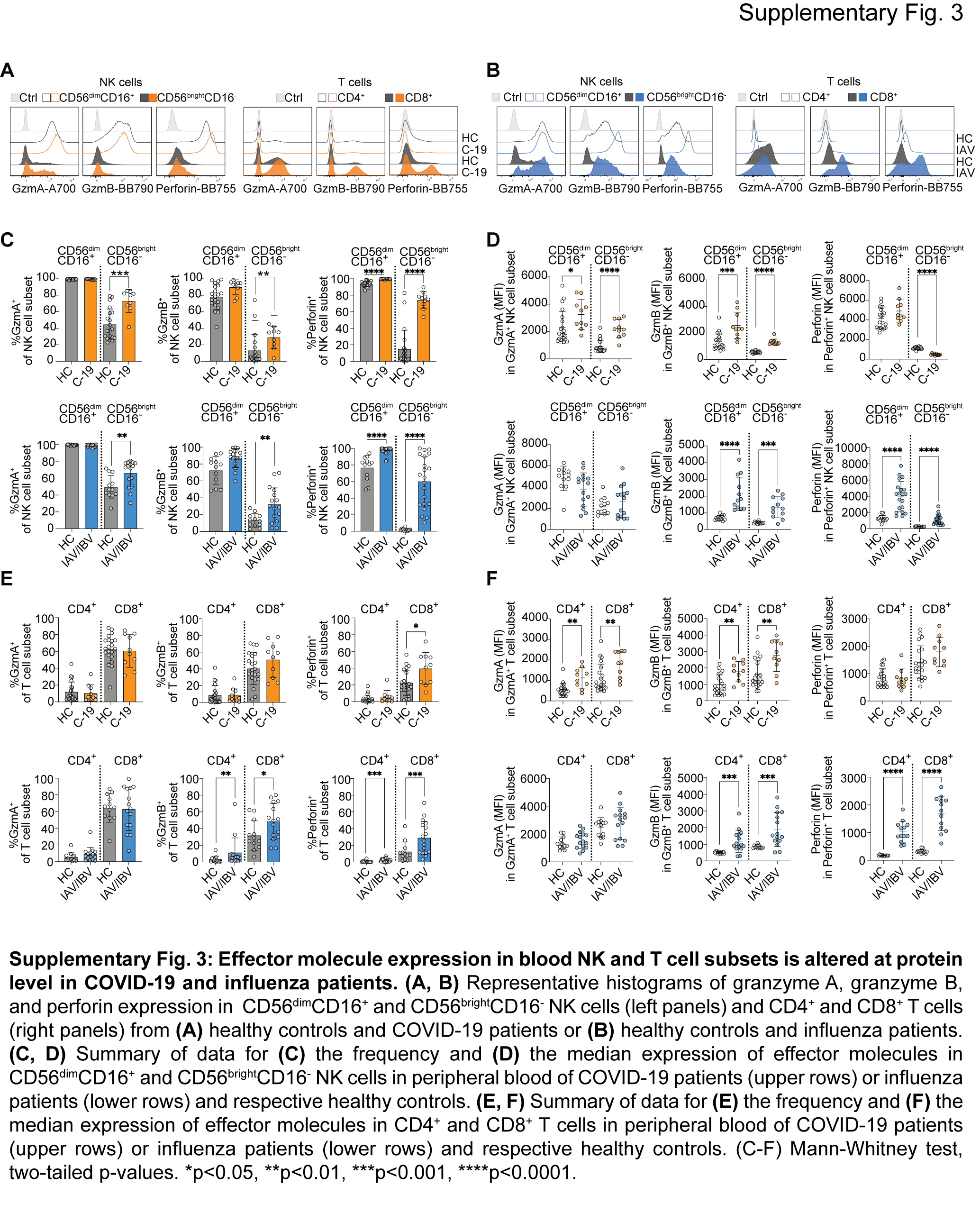

Supplement: Supplementary file 3 [file Image_3.tif]

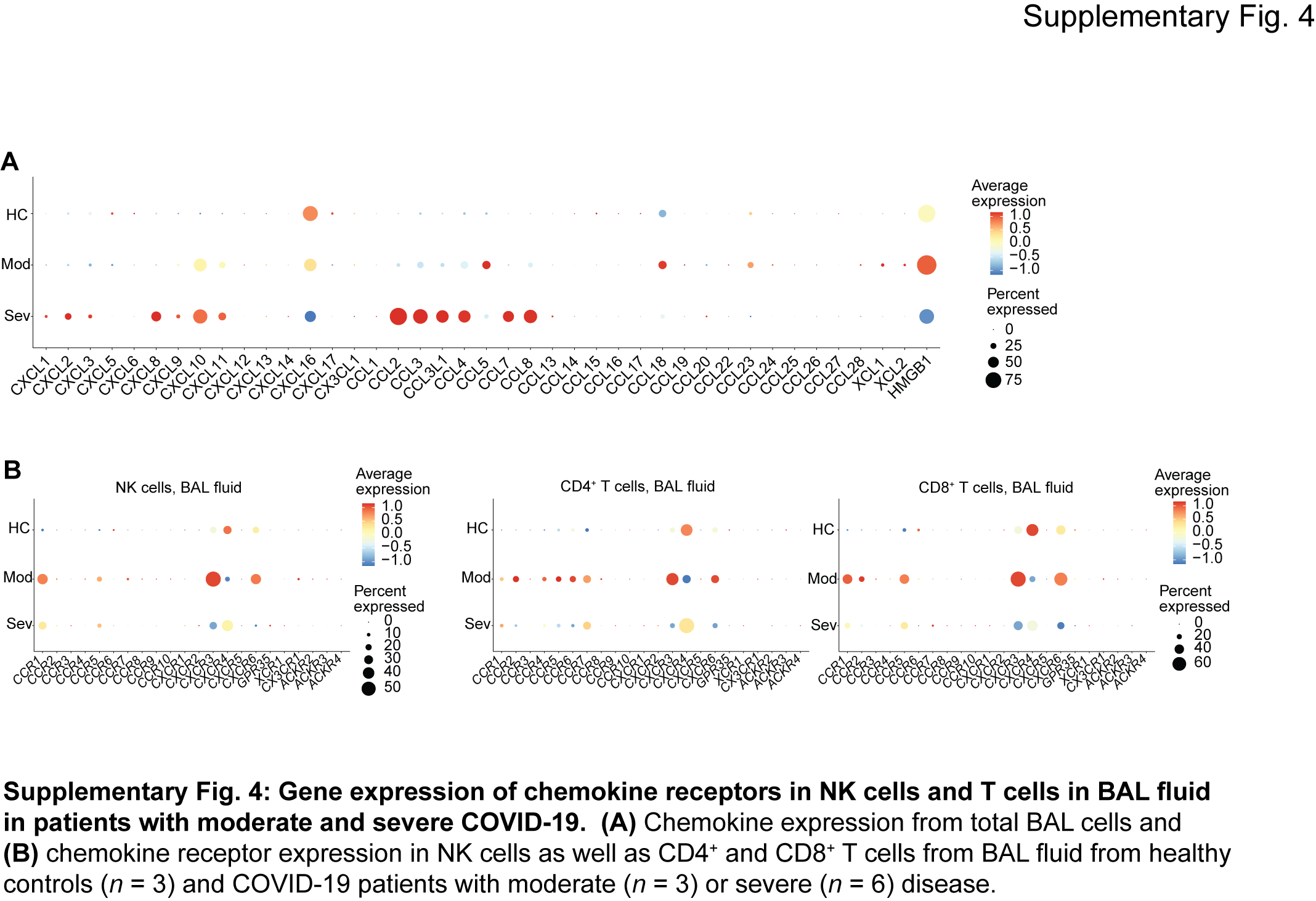

Supplement: Supplementary file 4 [file Image_4.tif]
