# Supplementary material for: Comparison of Lung-Homing Receptor Expression and Activation Profiles on NK Cell and T Cell Subsets in COVID-19 and Influenza
Source: Front Immunol. 2022 Mar 16;13:834862. doi: 10.3389/fimmu.2022.834862 (PMC8966396; doi:10.3389/fimmu.2022.834862)
Supplement: Supplementary file 5 [file Table_1.docx]

| **Antibody**  **Supplementary Table 1**. Antibodies used for phenotyping and intracellular staining. | **Clone** | **Fluorochrome(s)** | **Manufacturer** |
| --- | --- | --- | --- |
| CCR2 | K036C2 | BV711 | Biolegend |
| CCR5 | 3A9 | BV650 | BD Biosciences |
| CCR5 | J418F1 | BV785 | Biolegend |
| CCR7 | G043H7 | APC/Cy7 | Biolegend |
| CD3 | UCHT1 | PE/Cy5 | Beckman Coulter |
| CD3 | UCHT1 | BUV805 | BD Biosciences |
| CD4 | RPA-T4 | BV570 | Biolegend |
| CD8 | RPA-T8 | BUV395 | BD Biosciences |
| CD8 | SK1 | BUV737 | BD Biosciences |
| CD14 | MφP9 | Horizon V500 | BD Biosciences |
| CD16 | 3G8 | BUV496 | Biolegend |
| CD19 | HIB19 | Horizon V500 | BD Biosciences |
| CD38 | HIT2 | BUV661 | BD Biosciences |
| CD45RA | HI100 | BV785 | Biolegend |
| CD56 | NCAM16.2 | BUV563 | BD Biosciences |
| CD69 | FN50 | PE/CF594, BV750 | BD Biosciences |
| CXCR2 | 6C6 | BUV737 | BD Biosciences |
| CXCR3 | G025H7 | Alexa Fluor 647 | Biolegend |
| CXCR6 | K041E5 | BV421 | Biolegend |
| KIR2DL2/3/S2 | GL183 | PE/Cy5.5 | Beckman Coulter |
| KIR2DL1/S1 | EB6 | PE/Cy5.5 | Beckman Coulter |
|  | | | |
| Intracellular staining: | | | |
| **Antibody** | **Clone** | **Fluorochrome(s)** | **Manufacturer** |
| Granzyme A | CB9 | Alexa Fluor 700 | Biolegend |
| Granzyme B | GB11 | BB790 | BD Biosciences |
| Ki67 | B56 | BB660, Alexa Fluor 700 | BD Biosciences |
| Perforin | δG9 | BB755 | BD Biosciences |
